# Probiotic Supplementation and Inflammatory Status in Coronary Artery Disease: A Systematic Review and Meta-Analysis

**DOI:** 10.3390/microorganisms13102303

**Published:** 2025-10-04

**Authors:** Yuan-Yow Chiou, Tsu-Yun Chiu, Mei-Ju Chen

**Affiliations:** 1 Department of Pediatrics, Division of Pediatric Nephrology, National Cheng Kung University Hospital, College of Medicine, National Cheng Kung University, Tainan 704302, Taiwan; 2 Department of Psychology, Kaohsiung Medical University, Kaohsiung 807378, Taiwan; 3 Department of Senior Welfare and Services, Southern Taiwan University of Science and Technology, Tainan 710301, Taiwan

**Keywords:** C-reactive protein (CRP), coronary artery disease (CAD), inflammation, probiotics, systematic review and meta-analysis

## Abstract

Coronary artery disease (CAD), a major contributor to healthcare burdens worldwide, is closely linked with chronic inflammation. Probiotic supplementation has been investigated for its potential to modulate inflammatory responses, yet its role in patients with CAD remains unclear. To address this, we conducted a systematic review and meta-analysis following PRISMA guidelines, with literature searches performed across PubMed, EMBASE, and Cochrane CENTRAL up to 19 March 2025. Eligible studies included randomized controlled trials that examined the effects of probiotics, prebiotics, or synbiotics in patients with CAD or ischemic heart disease. Study quality was assessed using the Cochrane Collaboration tool, and standardized mean differences (SMDs) with 95% confidence intervals (CIs) were calculated for pooled outcomes. A total of five randomized controlled trials involving 256 patients with CAD were included. The meta-analysis demonstrated significant improvements in inflammatory biomarkers among participants receiving probiotics compared with those in the placebo group. Specifically, probiotic supplementation led to greater reductions in high-sensitivity C-reactive protein (pooled SMD [pSMD] = −0.61; 95% confidence interval [CI]: −0.87 to −0.36) and malondialdehyde (pSMD = −0.52; 95% CI: −0.91 to −0.12). No significant increase was observed in nitric oxide (pSMD = 0.91; 95% CI: −3.72 to 5.54) or total antioxidant capacity (pSMD = 0.35; 95% CI: −2.16 to 2.86) in the intervention group over control. No significant difference was found in glutathione levels between the two groups (pSMD = 0.01; 95% CI: −0.51 to 0.53). Overall, these findings suggest that probiotic supplementation exerts a beneficial effect on inflammatory status in patients with CAD. The evidence highlights its potential in reducing systemic inflammation and oxidative stress, as reflected by improvements in high-sensitivity C-reactive protein and malondialdehyde.

## 1. Introduction

Probiotics, comprising live microorganisms conferring health benefits upon their host and consumed fermented foods (e.g., yogurt, kefir, sauerkraut) or supplements, have gained attention for their potential to mitigate inflammation and modulate the immune system [1,2,3]. These beneficial bacteria have been studied extensively for their capacity to mitigate inflammation in diverse health conditions with a good safety profile. For instance, *Lactobacillus rhamnosus* and *Bifidobacterium longum* suppress pro-inflammatory cytokines, whereas *Lactobacillus plantarum* and *Lactobacillus casei* support gut-associated lymphoid tissue regulation [4,5,6]. One of the well-known plausible pathways is the gut–brain axis, through which probiotics regulate inflammation [3]. Specifically, the gut flora interacts with immune cells by influencing neurotransmitter synthesis (e.g., cytokine) and facilitating information exchange between the gut and the brain. Furthermore, probiotics produce short-chain fatty acids (SCFAs) like acetate, propionate, and butyrate, which play a crucial role in mitigating cardiometabolic risk and strengthening intestinal barriers [3,7]. Overall, probiotics have the potential to address inflammation and metabolism issues through multiple biochemical pathways.

Coronary artery disease (CAD), significantly influenced by chronic inflammation, constitutes a significant public health issue [8], being a leading cause of morbidity and mortality worldwide. Annually, CAD is associated with approximately 610,000 deaths in the United States, accounting for an estimated one-quarter of all US fatalities and being the nation’s lead cause of death. On a global scale, CAD stands as the third leading cause of death, contributing to roughly 17.8 million deaths annually. The annual healthcare expenditures attributed to CAD exceed USD 200 billion in the United States, signifying a significant economic impact within the healthcare infrastructure dedicated to managing this condition [9]. In addition, the Global Burden of Disease 2019 study projected a 91.2% increase in crude cardiovascular mortality in Asia by 2050, with East Asia, South Asia, and Southeast Asia experiencing the largest increases (147.4%, 85.3%, and 81.6%, respectively). Risk factors contributing to CAD extend beyond non-modifiable elements, such as age, sex, genetic predisposition, and family history. Modifiable factors, including diet, smoking, hypertension, dyslipidemia, diabetes mellitus (DM), obesity, and sedentary lifestyles, significantly influence an individual’s susceptibility to this disease [3,7,10,11].

CAD manifests through the gradual buildup of plaque within the coronary arteries. This process, coupled with chronic inflammation of initiation and progression of atherosclerosis, stands as a pivotal factor influencing the progression and complications of this condition, as supported by research findings [12,13]. Consequently, the concept of addressing inflammation to mitigate both mortality and morbidity associated with CAD has garnered extensive research and robust support [14,15]. On top of this, the effects of probiotic supplementation on inflammatory status in CAD remain unclear, so a comprehensive evaluation is warranted.

To the best of our knowledge, no prior meta-analysis has yet evaluated the effects of probiotic supplementary on inflammatory status in patients with CAD. Therefore, this systematic review and meta-analysis aims to review the existing literature and to synthesize evidence. It fills a critical gap in the medical literature and could offer deeper insights into the role of probiotics in cardiovascular health.

## 2. Methods

### 2.1. Search Strategy and Ethics Statement

The current systematic review and meta-analysis adhered to the Preferred Reporting Items for Systematic Reviews and Meta-Analyses (PRISMA) guidelines [16]. We conducted a comprehensive literature search across prominent public databases, such as PubMed, EMBASE, and Cochrane CENTRAL, employing the specific keywords “probiotics”, “prebiotic”, “synbiotics”, “coronary artery disease”, and “ischemic heart disease”, combined with Boolean operators, and by using Medical Subject-Headings (MeSH) terms where appropriate for studies published up to 19 March 2025. The search string used for the aforementioned databases is recorded in Supplementary File S1. Additionally, we conducted a manual search of the reference lists in the included studies to identify any potentially relevant research.

The systematic review and meta-analysis conducted in this study did not utilize raw patient data or private information. As a result, there was no requirement for further approval or informed consent from study subjects.

### 2.2. Selection Criteria

This review was conducted following the PICOS criteria, which encompass participants, interventions, comparisons, outcomes, and study design. Eligible patients were adults with CAD. The intervention of interest involved probiotic, prebiotic, or synbiotic supplementation, and the comparison group comprised individuals receiving no active treatment or a placebo. Outcomes of interest included studies reporting pre-treatment, post-treatment, or changes in inflammatory biomarkers. The eligible studies’ designs could be randomized clinical trials, prospective or retrospective cohort studies, or case–control studies.

We excluded review articles, letters, commentaries, editorials, proceeding research, conference abstracts, case reports, personal communications, and non-English studies.

The eligibility of each study was confirmed by two independent reviewers (Chiou, Y.-Y., and Chiu, T.-Y.), with a third reviewer (Chen, M.-J.) consulted for uncertain cases. This systematic review and meta-analysis is registered in the International Prospective Register of Systematic Reviews (PROSPERO) under the registration number CRD42024607040.

### 2.3. Main Outcome Measures and Data Extraction

Primary outcomes focused on changes in pre-treatment and post-treatment inflammatory biomarkers, including C-reactive protein (CRP), high-sensitivity CRP (hs-CRP), nitric oxide (NO), total antioxidant capacity (TAC), malondialdehyde (MDA), and glutathione (GSH) [12,17,18,19].

From the eligible studies, we extracted the following information: the first author’s name, publication year, study design, number of patients in each group, diagnosis, patients’ age and sex (male), type of probiotics, dosage, duration of intervention, and inflammatory biomarkers of interest.

### 2.4. Quality Assessment

The quality of the included studies was assessed using the Cochrane Collaboration tool [20]. This tool assesses risk of bias via the following seven criteria: selection bias (random sequence generation and allocation concealment), performance bias (blinding of participants and personnel), detection bias (blinding of outcome assessment), attrition bias (incomplete outcome), reporting bias (selective outcome reporting), and inclusion of intention-to-treat analysis. Two independent reviewers conducted the quality assessment (Chiou, Y.-Y., and Chiu, T.-Y.) and a third reviewer (Chen, M.-J.) was consulted to resolve any uncertainties.

### 2.5. Statistical Analysis

The outcomes were evaluated using the standardized mean difference (SMD) along with 95% confidence intervals (CI). Heterogeneity among the studies was evaluated using the Cochran Q test and I^2^ statistic [21]. Heterogeneity was defined as follows: I^2^ ≤ 25%, low heterogeneity; 25% < I^2^ < 50%, moderate heterogeneity; 50% ≤ I^2^ < 75%, substantial heterogeneity; and I^2^ ≥ 75%, high heterogeneity. If I^2^ was greater than 50%, evidence of substantial-to-high heterogeneity was indicated, and random effects models were employed; otherwise, fixed effects models were used. For outcomes with ≤4 studies, a random effects model was used irrespective of I^2^ < 50%. In this meta-analysis, we refrained from analyzing publication bias due to the limited number of studies included (fewer than 10 for each outcome) [22]. We used a two-sided test with a significance level of α = 0.05 for statistical analysis. All analyses were conducted using R Studio 4.3.2 with the packages “meta”, “dmetar”, and “metafor”.

## 3. Results

### 3.1. Study Selection

**Figure 1:**
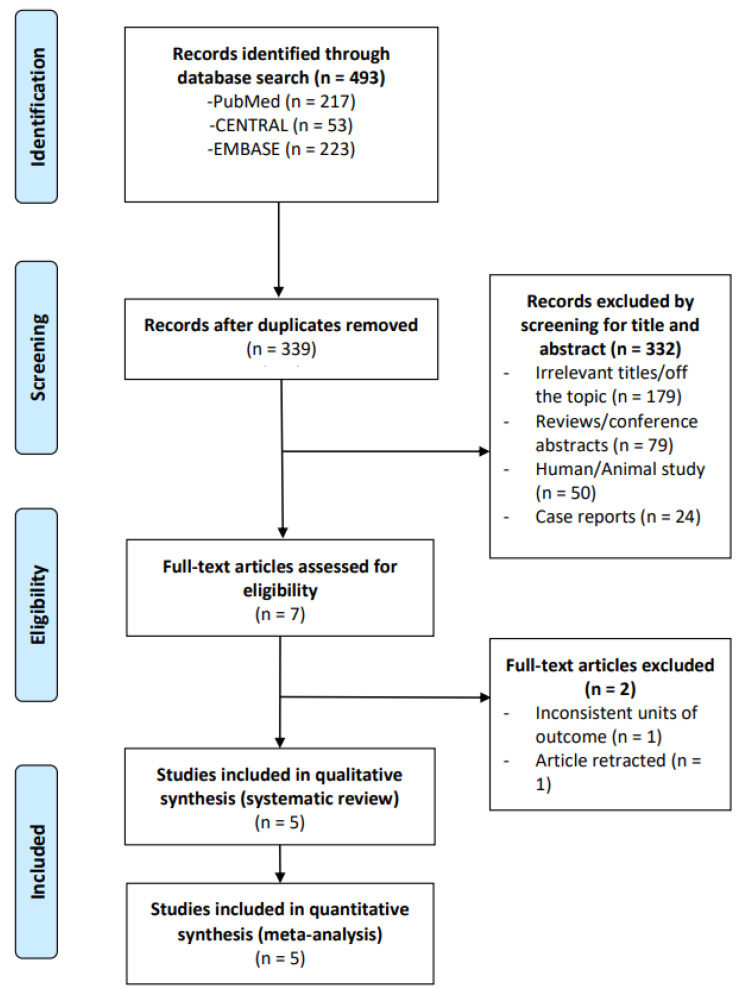
PRISMA flow diagram of study selection.

The flow diagram of the study selection process is demonstrated in Figure 1. A total of ten full-text articles were assessed for eligibility, but assessment excluded five of these studies. This left five studies [23,24,25,26,27] to be included in both the qualitative and quantitative synthesis (Figure 1). The five included studies encompassed a total of 256 patients.

The number of search hits corresponding to each step of the systematic literature search, qualitative review, and quantitative analysis are shown. The reasons for search hit exclusion are described.

### 3.2. Characteristics of the Included Studies

The study characteristics are summarized in Table 1 and Supplementary File S2. All studies were RCTs. The mean age ranged from 52 to 69 years. The proportion of male patients ranged from 37% to 93%, and female patients from 7% to 63%. The duration of probiotic treatment ranged from 8 weeks to 12 weeks. Moreover, two studies regarding synbiotics were included, and the others were probiotic-related. No included study focused only on prebiotics. Among the included trials, the most frequently used probiotic strain was *Lactobacillus rhamnosus*. One study using the commercial gluten-free product Lactocare^®^ employed a multi-strain formulation containing *Lactobacillus rhamnosus*, *Lactobacillus casei*, *Lactobacillus acidophilus*, *Bifidobacterium breve*, *Lactobacillus bulgaricus*, *Bifidobacterium longum*, and *Streptococcus thermophilus*. In addition, the product contained fructo-oligosaccharides as a prebiotic to promote the growth and activity of beneficial bacteria.

### 3.3. Quality Assessment (Risk of Bias)

**Figure 2:**
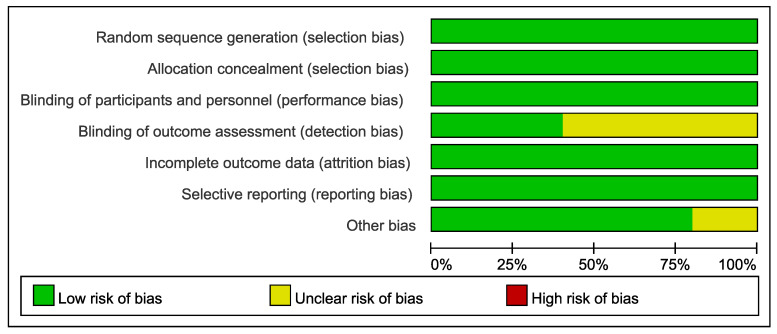
Risk of bias graph.

**Figure 3:**
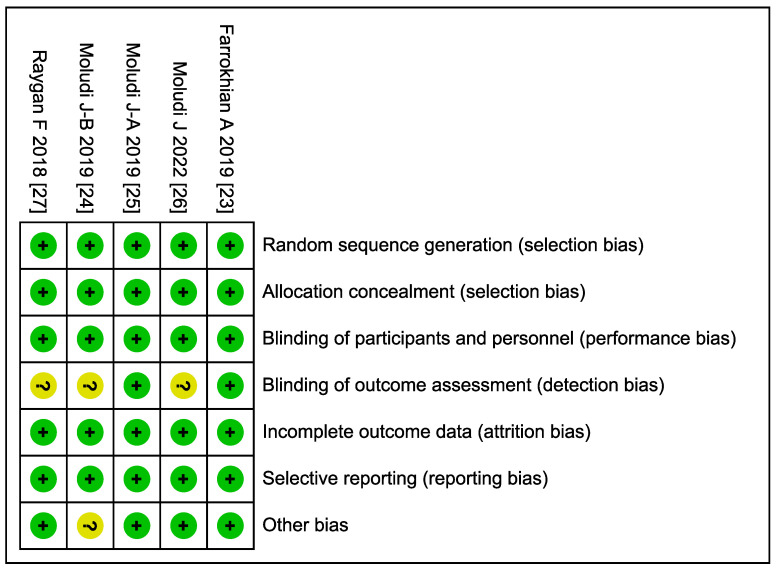
Risk of bias summary [23,24,25,26,27].

The methodological quality of the included RCTs was evaluated using the Cochrane Risk of Bias tool. As shown in Figure 2, most studies demonstrated a low risk of bias across domains. Specifically, all trials were assessed as low-risk for random sequence generation, allocation concealment, and incomplete outcome data. The majority also showed low risk in blinding of participants and personnel, as well as in selective reporting. However, there was some uncertainty in the blinding of outcome assessment and selective reporting in a minority of studies. Notably, a small proportion of studies were rated as having unclear risk in the domain of other bias. Figure 3 further illustrates that while the majority of studies (e.g., Farrokhian A 2019 [23], Moludi J 2022 [26], and Raygan F 2018 [27]) consistently met low-risk criteria, certain studies (e.g., Moludi J-B 2019 [24]) had unclear risk ratings in specific domains such as selective reporting and performance bias (Supplementary File S2).

### 3.4. Meta-Analysis

**Figure 4:**
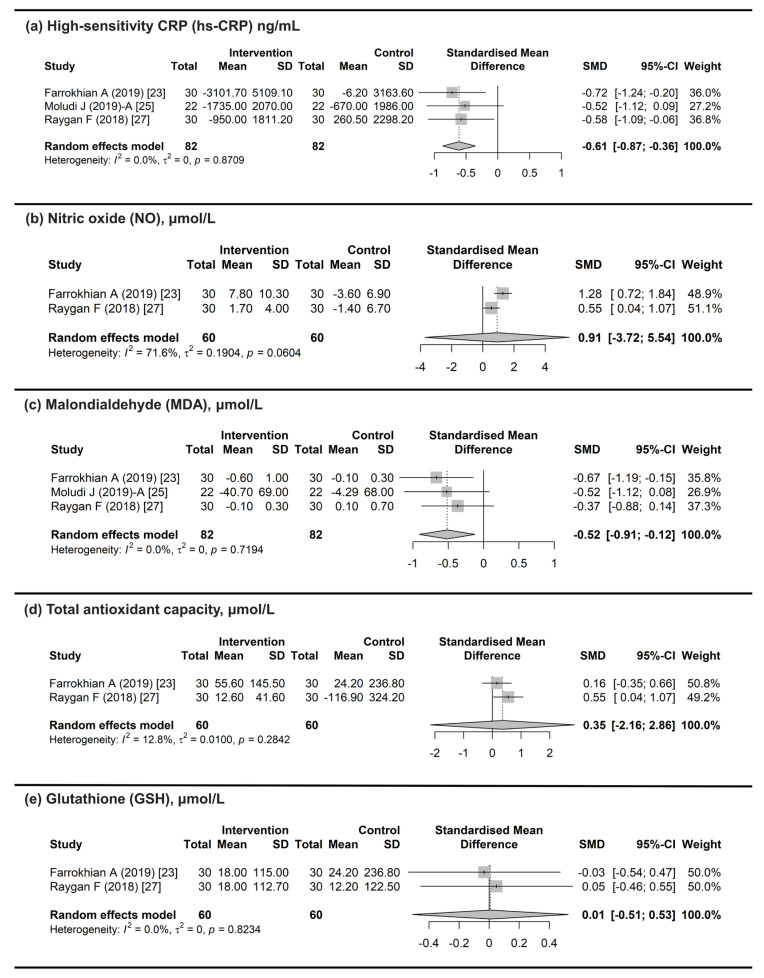
Differences in changes in inflammatory and antioxidant markers between probiotics and control groups [23,25,27]. (**a**) hs-CRP: Significantly greater improvement in hs-CRP in the probiotics group than in the control (pooled SMD = −0.61; 95% CI: −0.87 to −0.36) using a random effects model (I^2^ = 0%). (**b**) NO: No significant difference in the change in NO observed between the probiotic and control groups (pooled SMD = 0.91; 95% CI: −3.72 to 5.54) using a random effects model (I^2^ = 71.6%). (**c**) MDA: Significantly greater improvement in MDA in the probiotics group than in the control (pooled SMD = −0.52; 95% CI: −0.91 to −0.12) using a random effects model (I^2^ = 0%). (**d**) TAC: No significant difference in the change in TAC observed between the probiotic and control groups (pooled SMD = 0.35; 95% CI: −2.16 to 2.86) using a random effects model (I^2^ = 12.8%). (**e**) GSH: Significantly greater improvement in GSH in the probiotics group than in the control (pooled SMD = 0.01; 95% CI: −0.51 to 0.53) using a random effects model (I^2^ = 0%). Abbreviations: hs-CRP, high-sensitivity C-reactive protein; NO, nitric oxide; MDA, malondialdehyde; TAC, total antioxidant capacity; GSH, glutathione; SMD, standardized mean difference; CI, confidence interval.

For the change in hs-CRP, three studies [23,25,27] were included in the meta-analysis. Significant mean differences in the change in hs-CRP were observed (pooled SMD = −0.61; 95% CI: −0.87 to −0.36) utilizing the random effects model (I^2^ = 0%). This analysis suggests that the probiotics group had a significantly greater improvement compared to the control group (Figure 4a).

For the change in NO, two studies [23,27] were included in the meta-analysis. There were no significant mean differences in the change in NO (pooled SMD = 0.91; 95% CI: −3.72 to 5.54) under the random effects model (I^2^ = 71.6%) (Figure 4b).

For the change in MDA, three studies [23,25,27] were included in the meta-analysis. Significant mean differences in the change in MDA were observed (pooled SMD = −0.52; 95% CI: −0.91 to −0.12) using the random effects model (I^2^ = 0%). This analysis suggests that the probiotics group had a significantly greater improvement compared with the control group (Figure 4c).

For the change in TAC, two studies [23,27] were included in the meta-analysis. There were no significant mean differences in the change in TAC (pooled SMD = 0.35; 95% CI: −2.16 to 2.86) using the random effects model (I^2^ = 12.8%) (Figure 4d).

For the change in GSH, two studies [23,27] were included in the meta-analysis. Significant mean differences in the change in GSH were observed (pooled SMD = 0.01; 95% CI: −0.51 to 0.53) using the random effects model (I^2^ = 0%). This analysis suggests that the probiotics group had a significantly greater improvement compared with the control group (Figure 4e).

## 4. Discussion

CAD poses a significant global health burden, as it is associated with elevated morbidity and mortality throughout the world [8,9]. Despite advances in treatment modalities, CAD remains a leading cause of death, so exploration of novel therapeutic strategies is warranted. Such exploration should focus on inflammation, a process that is thought to play a pivotal role, both in the pathogenesis of atherosclerotic cardiovascular disease, including CAD, and in the process that generates thrombosis within atherosclerotic vessels, leading to acute coronary syndrome [12,13]. The present meta-analysis is the first in the literature to evaluate the impact of probiotic supplementation on inflammatory status in patients with CAD. The results indicated a significantly greater improvement in inflammatory biomarkers in the probiotics treatment group over the control group, namely, a greater reduction in hs-CRP and MDA. Nevertheless, changes in NO, TAC, or GSH were not significantly different between the two groups. Heterogeneity was low across the outcomes except for the analysis of NO, indicating the robustness of the analyses. These findings point toward the potential of probiotic consumption to decrease inflammation in the context of CAD.

Beyond RCTs, observational human studies lend additional support. A recent large cross-sectional analysis of NHANES data (1999–2019) reported that CAD patients who regularly consumed probiotics had a more favorable cardiovascular biomarker profile—including lower triglycerides, HbA1c, and a lower estimated 10-year atherosclerotic cardiovascular disease risk—compared to those not consuming probiotics [28]. Although direct inflammatory markers (e.g., CRP) were not highlighted in that analysis, the results imply an overall healthier metabolic and inflammatory state in probiotic users. This association must be interpreted with caution due to potential confounding factors (healthier individuals may be more likely to take probiotics). Nonetheless, it aligns with the concept that gut microbiota modulation is linked to improved systemic health in CAD. In acute coronary settings, emerging data suggest a similar trend: one study observed that higher intestinal *Lactobacillus* abundance in patients with acute myocardial infarction correlated with a reduced inflammatory response and better short-term prognosis [29]. Taken together, both interventional and observational studies reinforce the notion that targeting the gut microbiome—especially with *Lactobacillus* probiotics—can beneficially influence the inflammation in coronary disease.

The ability of *Lactobacillus* strains to attenuate inflammation in CAD is underpinned by several biological mechanisms. A primary mode of action is through microbiota remodeling. Probiotic *Lactobacilli* can favorably alter gut microbiome composition by increasing beneficial commensals and suppressing pro-inflammatory pathobionts. Metagenomic analyses have shown that atherosclerosis patients tend to harbor gut microbiomes enriched in pro-inflammatory and lipopolysaccharide (LPS)-producing pathways and depleted in anti-inflammatory metabolic pathways (such as those generating short-chain fatty acids) [30]. Introducing beneficial bacteria like Lactobacilli may counteract this dysbiosis. Indeed, 16S rRNA sequencing in the *L. plantarum* 299v trial confirmed an enrichment of the *Lactobacillus* genus in the gut after supplementation [30], and functional readouts showed increased production of the SCFA propionate [30]. SCFAs have well-documented anti-inflammatory properties: propionate and butyrate engage G protein-coupled receptors and promote regulatory immune responses (e.g., inducing IL-10 and T_regs) while suppressing NF-κB signaling in immune cells. The rise in propionate observed with L. plantarum [30] suggests that enhanced SCFA production may contribute to the reduced systemic inflammation and improved endothelial function noted in certain patients [31]. In parallel, probiotics can produce other metabolites (e.g., tryptophan catabolites and conjugated linoleic acids) that modulate immune cell activity and may reduce pro-inflammatory cytokine release, though specific data in CAD patients are still emerging.

Another critical mechanism is the strengthening of gut barrier integrity. *Lactobacillus* probiotics have been shown to fortify tight junction proteins and mucous layer thickness, thereby reducing intestinal permeability (“leaky gut”) and translocation of endotoxin into circulation [32,33]. This is evidenced by significant reductions in serum LPS concentrations in probiotic-treated groups. In the LGG trial, LPS fell markedly in the probiotic arm versus a placebo [33], and a combination therapy using an inulin prebiotic plus a *Lactobacillus* probiotic led to a ~30% drop in LPS alongside down-regulation of Toll-like receptor 4 (TLR4) expression in CAD patients [32]. By blunting systemic endotoxemia, *Lactobacilli* likely interrupts a key driver of inflammation: LPS-induced monocyte activation. Reduced LPS–TLR4 signaling translates into the lower downstream release of IL-6, IL-1β, and other cytokines, as reflected by the significant IL-6 reductions observed with synbiotic (probiotic + prebiotic) therapy in a recent trial [32]. This gut barrier mechanism is pivotal since chronic exposure to microbial LPS is thought to sustain the vascular inflammation underlying atherosclerosis. The improvement in metabolic endotoxemia with probiotics (sometimes by over 50% reduction in LPS levels [34]) is therefore a compelling explanation for the observed anti-inflammatory clinical effects.

Modulation of microbiota-derived metabolites provides additional insight, particularly regarding the Trimethylamine-N-oxide (TMAO) pathway. TMAO, produced from dietary nutrients by gut bacteria, has emerged as an inflammatory mediator linked to atherosclerotic risk. Some *Lactobacillus* species can impact this pathway. A 2022 systematic review identified *L. rhamnosus GG* as one of the most effective strains for lowering plasma TMAO in both humans and animals [35]. The proposed mechanisms included competition with, or inhibition of, TMA-generating gut microbes and perhaps enhanced utilization of dietary choline/carnitine by alternate (non-TMA-producing) pathways. Lowering TMAO may attenuate macrophage activation in plaques and reduce foam cell formation and cytokine release. However, evidence on probiotics and TMAO in CAD remains mixed. In Malik et al.’s trial, *L. plantarum* 299v did not significantly change TMAO levels over 6 weeks [36], even though it improved other inflammatory indices. This discrepancy highlights that strain specificity and treatment duration are important—not all *Lactobacilli* exert the same effects on TMAO, and longer interventions or multi-strain combinations may be required to meaningfully influence this metabolite. Nonetheless, the ability of certain *Lactobacillus* strains to favorably modulate TMAO (as well as related metabolites like trimethylamine or bile acids) remains an exciting avenue by which gut flora interventions could mitigate CAD-related inflammation.

In addition to these microbiome-mediated pathways, *Lactobacillus* probiotics may directly influence host inflammatory and oxidative stress pathways. Many *Lactobacilli* produce antioxidant molecules (e.g., glutathione, peroxidases) or induce host antioxidant enzymes, thereby reducing oxidative stress in tissues. Oxidative stress and inflammation are intrinsically linked in atherosclerosis, as oxidative modifications (e.g., low-density lipoprotein oxidation) trigger inflammatory immune responses. Clinical studies have documented improvements in oxidative biomarkers with probiotic therapy—for example, decreased MDA, a lipid peroxidation marker, and elevated TAC and GSH levels [32]. A recent meta-analysis of trials in patients with cardiometabolic disorders found that probiotic/synbiotic use led to significant increases in TAC and GSH, reflecting an overall enhancement in antioxidant defenses [32]. By quelling oxidative stress, *Lactobacilli* may indirectly dampen inflammatory signaling (since fewer reactive oxygen species are available to activate redox-sensitive inflammatory pathways). Furthermore, components of *Lactobacillus* cell walls (such as peptidoglycans and surface-layer proteins) can interact with pattern recognition receptors on immune cells in the gut mucosa, biasing cytokine production toward an anti-inflammatory profile. This is supported by observations of increased IL-10 (an anti-inflammatory cytokine) and expansion of regulatory T-cells in some probiotic studies [30]. Such immune system modulation suggests that probiotics not only reduce pro-inflammatory triggers but also actively enhance anti-inflammatory mediators.

In the context of CAD pathogenesis, inflammation is thought to involve migration of CD4+ T lymphocytes into atherosclerotic plaques, which would trigger the expression of pro-inflammatory, TH1-associated cytokines [12,37]. Notably, these cytokines include IFN-γ, IL-2, IL-3, TNF, and lymphotoxin, which, in turn, can activate macrophages [37]. Importantly, cytokines, such as IL-1β, IL-6, and IL-8, are known to potentiate both primary hemostasis (formation of platelet plugs) and secondary hemostasis (the coagulation cascade) [38], which, in the setting of CAD, can explain myocardial infarction [12].

In addition, older adults have elevated cardiovascular risk, microbiota dysbiosis, and increased oxidative stress, a combination that promotes ‘inflammaging’ and endothelial dysfunction [39]. Sustained probiotic intake may help restore beneficial microbes and gut barrier integrity, lowering endotoxemia and inflammation. Combined with antioxidants, this could better counteract oxidative stress and preserve vascular health. In the long term, this dual strategy holds promise as an adjunctive approach to reduce CAD risk in aging populations.

Antibiotic exposure is a significant confounder that can disrupt gut microbiota, reducing SCFA producers and favoring pro-inflammatory taxa [40]. This dysbiosis may worsen systemic inflammation and offset probiotic benefits. Notably, the included RCTs rarely reported antibiotic use in study participants, which limits interpretation. Future studies should control for recent antibiotic exposure to better isolate the effect of probiotic supplementation on inflammatory outcomes in CAD patients.

Our analysis primarily focused on hs-CRP and MDA, which were the most frequently assessed markers in the studies included. hs-CRP is produced by hepatocytes in response to inflammatory cytokines [12,31]. MDA not only is a marker for systemic inflammation but has also been useful as an inflammatory marker related to coronary stress [17]. Additionally, NO levels were included in the analysis because of the role of NO in vasodilation [18] and because of its possible anti-inflammatory effects [19].

The mechanisms by which probiotics might reduce inflammation in coronary vascular endothelium remain largely hypothetical, though several plausible explanations warrant further investigation. One such mechanism involves probiotics attenuating systemic inflammation by offsetting dysbiosis among gut microbiota [2,4]. This process also encompasses reduction in endothelial dysfunction, but the specific steps in such a mechanism need to be well defined and studied at the basic science level [2].

Given the positive outcomes observed in our meta-analysis, it is imperative to consider the integration of probiotic supplementation into broader CAD management strategies. The consistent reduction in inflammatory markers such as hs-CRP and MDA suggests that probiotics could serve as an adjunctive therapy, potentially enhancing the efficacy of conventional treatments.

### 4.1. Strengths and Limitations

The current meta-analysis is novel as it is the first published study on the effect of probiotics on inflammatory parameters in the setting of CAD. This study furthermore only encompasses RCTs, which is an additional strength. Consistently narrow confidence intervals across pool effect sizes underscore the accuracy and reliability of the research outcomes, so heterogeneity is low in most of the outcomes.

To be sure, the current study also suffers from some limitations. Firstly, the limited number of included studies constrained the inclusion pool. Furthermore, diversity was restricted, as these few trials were conducted solely by three research teams within the same country. Although there were variations in probiotic type and dosage across the trials, the limited number of studies prevented subgroup analysis. Accordingly, future updated meta-analyses are encouraged to encompass a more extensive range of trials from different countries to enhance the generalizability of the findings.

### 4.2. Directions of Future Research

While the evidence for *Lactobacillus* probiotics mitigating inflammation in CAD is growing stronger, several considerations guide and temper the enthusiasm of future research. Firstly, trials to date have been relatively small in sample size and short in duration, primarily measuring surrogate endpoints (inflammatory biomarkers, endothelial function) rather than “hard” cardiovascular outcomes. It remains to be proven whether the reductions in CRP or IL-6 achieved with probiotics will translate into fewer cardiac events or slower atherosclerosis progression. Larger, long-term RCTs are warranted to confirm clinical benefits. Secondly, there is notable heterogeneity across studies in terms of strains used, dose regimens, and patient populations. Different *Lactobacillus* strains vary in their functional properties; for example, *L. rhamnosus* may excel at TMA/TMAO reduction [36], whereas *L. plantarum* may have stronger effects on endothelial function and chemokines [30]. Multi-strain formulations (often combining *Lactobacilli* with *Bifidobacteria*) have shown broad metabolic and inflammatory improvements [34], but identifying the contribution of each strain is challenging. Future studies should compare strains head-to-head and elucidate which specific microbes (or microbial metabolites) drive the greatest cardioprotective effects. Additionally, optimal dosing and treatment duration need clarification—some anti-inflammatory effects (e.g., reductions in IL-6 or TNF-α) might require longer exposure or higher CFU doses to manifest consistently.

An important practical insight from recent trials is the synergy between probiotics and diet. In Moludi et al.’s study, all participants were prescribed a calorie-restricted diet, and those who achieved ≥2.5 kg weight loss had improvements in inflammatory markers irrespective of probiotic use [33]. This underlines that lifestyle modification remains fundamental; probiotics should be viewed as an adjunct to, not a replacement for, standard risk factor management (diet, exercise, and medications). Notably, the greatest anti-inflammatory gains in one trial were seen when a prebiotic (inulin) was combined with a *Lactobacillus* probiotic, rather than either one alone [32]. Such findings suggest that fostering a supportive gut environment (through dietary fibers or prebiotics) may amplify the benefits of probiotic supplementation. They also raise questions about the influence of baseline diet, antibiotics, and co-medications (e.g., statins, which themselves have mild anti-inflammatory effects) on probiotic efficacy. In the *L. plantarum* FMD trial, a subgroup of patients with regular alcohol intake showed a blunted response to a probiotic [30], hinting that host factors and concomitant exposures can modulate outcomes. Personalizing probiotic therapy—selecting strains based on an individual’s microbiome composition or risk factor profile—could be a future strategy to maximize anti-inflammatory effects in CAD.

## 5. Conclusions

The findings of this systematic review and meta-analysis suggest that probiotic supplementation, observed across studies using various *Lactobacillus* and *Bifidobacterium* strains, significantly improves inflammatory status in individuals with CAD. These improvements include reductions in hs-CRP and MDA levels highlighting the potential of probiotics as a beneficial adjunct in managing CAD-related inflammation.

## Figures and Tables

**Table 1:** Characteristics of the included studies. Study design: RCT.

Study	Number of Patients	Mean Age (Years)	Sex (%)	Diagnosis	Type of Probiotic	Dosage (CFU)	Duration of Intervention (Weeks)
Moludi J (2022) [26]	Probiotic: 24 Placebo: 24	51.5	Female: 35.4 Male: 64.6	CAD	*Lactobacillus rhamnosus*	1.9 × 10^9^	8
Farrokhian A (2019) [23]	Synbiotic: 30 Placebo: 30	64.1	Female: 63.3 Male: 36.7	CAD with overweight and T2DM	*Lactobacillus acidophilus*; *Lactobacillus casei*; *Bifidobacterium bifidum*	2 × 10^9^	12
Moludi J (2019)-A [25]	Probiotic: 22 Placebo: 22	56.9	Female: 6.8 Male: 93.2	CAD with myocardial infarction	*Lactobacillus rhamnosus*	1.6 × 10^9^	12
Moludi J (2019)-B [24]	Probiotic: 22 Placebo: 22	52.6	Female: 6.8 Male: 93.2	CAD	*Lactobacillus rhamnosus*	1.6 × 10^9^	12
Raygan F (2018) [27]	Synbiotic: 30 Placebo: 30	69.4	Female: 50.0 Male: 50.0	2- and 3-vessel CAD with T2DM	Lactocare^®^ (ZistTakhmir Company)	8 × 10^9^	12

CAD, coronary artery disease; T2DM, type 2 diabetes mellitus; CFU, colony-forming unit; RCT, randomized control trial.

## Data Availability

The datasets analyzed during the current study are available from the corresponding author on reasonable request.

## Supplementary Materials

The following supporting information can be downloaded at https://www.mdpi.com/article/10.3390/microorganisms13102303/s1, Supplementary File S1: The searching strategy for the research. Supplementary File S2: Characteristics and risk of bias of the included studies. Supplementary File S3: PRISMA 2020 checklist of the research.
