# Supplementary material for: Probiotic Supplementation and Inflammatory Status in Coronary Artery Disease: A Systematic Review and Meta-Analysis
Source: Microorganisms. 2025 Oct 4;13(10):2303. doi: 10.3390/microorganisms13102303 (PMC12565933; doi:10.3390/microorganisms13102303)
Supplement: Supplementary file 1 [file microorganisms-13-02303-s001.zip › microorganisms-3634051_Supplementary file S1.pdf]

**Supplementary file 1**

**Search strategy:**

*((coronary artery disease) OR (ischemic heart disease) OR (acute coronary syndrome) OR (myocardial infarction) OR (Myocardial Ischemia))*

*AND*

*(Probiotics OR probiotic* OR prebiotics OR Bifidobacterium OR Bifido* OR Bacteroides OR Bacteroid* OR Lactobacillus OR Lactobacil* OR Lactobacillaceae OR Pediococcus OR (Fermented Foods and Beverages) OR Nissle OR (Fermented Foods) OR Streptococ* OR Saccharomyces OR Saccharomy OR Enterococcus OR Lactobacillales OR (Lactic acid bacteria) OR (Bacillus mesentericus) OR (Escherichia coli) OR*

*acidophilus OR microorganism* OR synbiotics OR Synbiotic* OR Symbiotic*)*

*AND*

*(random* controlled trial [pt] OR controlled clinical trial* [pt] OR randomized [tiab] OR placebo [tiab] OR drug therapy [sh] OR random* [tiab] OR trial* [tiab] OR group* [tiab] OR Clinical Trial, Phase III [pt] OR Cohort Studies OR Case-Control Studies)*

*AND*

*(Biomarkers OR inflammatory OR antioxidants OR (hs-CRP) OR (high-sensitivity C-reactive protein) OR (Nitric oxide) OR MDA OR Malondialdehyde OR TAC OR (Total antioxidant capacity) OR GSH OR Glutathione)*

*NOT*

*(animals [mh] NOT humans [mh])*
